# Gender difference in prevalence of hypertension among Indians across various age-groups: a report from multiple nationally representative samples

**DOI:** 10.1186/s12889-022-13949-5

**Published:** 2022-08-10

**Authors:** Parimala Mohanty, Lipilekha Patnaik, Gayatri Nayak, Ambarish Dutta

**Affiliations:** 1 Department of Community Medicine, Institute of Medical Sciences and Sum Hospital, Siksha ’O’ Anusandhan Deemed to be University, Bhubaneswar, India; 2 Indian Institute of Public Health, Public Health Foundation of India, Bhubaneswar, India; 3 Indian Institute of Public Health, Public Health Foundation of India, Address-Plot No- 267/3408, JaydevVihar, Mafair Lagoon Road, Odisha 751013 Bhubaneswar, India

**Keywords:** Hypertension, Gender, Age-groups, BMI, NFHS, LASI, SAGE, India

## Abstract

**Background:**

Prevalence of hypertension increases with age, but there is a general perception in India that women are less affected at every stage of life, although empiric evidence hardly exists regarding gender difference in hypertension in Indians of different ages. Therefore, we aimed to examine the gender difference in hypertension among Indians across various age-groups; and the contribution of variation in body mass index (BMI) to this difference.

**Methods:**

Data were analysed after combining National Family Health Survey 4 (*n* = 294,584 aged 35–49 years) and Study of Ageing and Health wave 2 (*n* = 7118 aged 50 + years) datasets (NFHS-SAGE). Longitudinal Ageing Study of India (LASI) dataset (*n* = 65,900 aged > 45years) was analysed to replicate the results. Hypertension was defined if systolic and diastolic blood pressure was > 89 and/or > 139 respectively and/or if there was a history of anti-hypertensive medication. Descriptive summaries were tabulated and plotted to examine the gender difference in hypertension in various age-groups (35–39,40–44, 45–49, 50–54, 55–59, 60–64, 65–69, ≥ 70). Odds Ratios (ORs) from logistic regression models estimated the age gradient of hypertension and their male-female difference, adjusted for Body Mass Index (BMI).

**Results:**

Males had a higher prevalence of hypertension up to 50 years; after that, females had higher rates. The estimates of age gradient, expressed as ORs, were 1.02 (1.02, 1.02) in males versus 1.05(1.05, 1.06) in females (*p* < 0.001) in NFHS-SAGE and 1.01(1.01, 1.02) in males versus 1.04(1.03, 1.04)in females (*p* < 0.001) in LASI;these differences marginally changed after adjustment with BMI.

**Conclusion:**

This is perhaps the first study to comprehensively demonstrate that cardio-metabolic risk in Indian females surpasses males after 50 years of age, “busting the myth” that Indian females are always at much lower risk than males; and this evidence should inform the Indian healthcare system to prioritise older women for screening and treatment of hypertension.

**Supplementary Information:**

The online version contains supplementary material available at 10.1186/s12889-022-13949-5.

## Introduction

Across the globe, Non-communicable diseases (NCD) are the leading preventable causes of mortality [1]. Among NCDs and their risk factors, hypertension has long been recognized as a leading cause of morbidity and premature death [2] with an estimated 1.13 billion people worldwide suffering from it [3].

Recently, the low- and middle-income countries (LMICs) have also been experiencing a rapid increase in the prevalence of hypertension, which had gone up from 599 million in 2000 to 1.0 billion in 2010 in those nations [4]. This is possibly driven by rapid urbanization of LMICs, ageing of their populations, changes in their dietary habits and increase in social stress in those economically emerging societies [5, 6]. In India, a substantial proportion (29.8% as per recent estimates) of adults suffer from hypertension [3, 7–9].

Meanwhile, a popular perception persists worldwide that females are less prone to cardiovascular diseases (CVD) including hypertension [10–12] and these are basically “man’s problems” [13]. This has led even females to underestimate their CVD risks [14]. Even more worrying, physicians being swayed by this also tend to under-test and under-treat females for hypertension and other cardiovascular pathologies [15]. As per anecdotal evidence this perception of females being less prone to hypertension and CVD than males are also rife in India.

However, evidence shows that these CVD-related health conditions are not only widely prevalent in females, but also in later adult life females suffer from more hypertension than males, albeit males experience a higher prevalence than females at a younger age [16–18]. This pattern has been attributed partly to biological causes, and partly to gender differences in socio-behavioural factors, for example overweight and health care access [19]. But, the body of evidence regarding male-female difference in hypertension and their changing patterns in different stages of life has some significant gaps. First, it is almost entirely from high income nations, and hardly from any LMICs, though LMICs may experience a different gender pattern given the variations in gender-related social norms and behaviours between these cultures [20–24]. Second, the evidence is also somewhat nebulous in nature, because there is inconsistency in literature about the reversal of the gender pattern and the age at which it occurs [23, 25–27].

As mentioned above, there is hardly any comprehensive empiric evidence of the gender differences in CVD and its risk factors such as hypertension among Indians in different stages of their adult lives. Until now, Indian studies analysing age-pooled data have mainly reported male dominance in the overall prevalence of hypertension [27–29]. The gender difference in different ages have been reported by only one Indian study to our knowledge, which also showed greater rates in males in all age groups [7], and no sign of reversal, contrary to evidence from high income nations. Hence, this domain urgently warrants robust India-specific evidence so that any prevailing gender stereotype can be challenged.

Therefore, we primarily aimed to examine gender difference in hypertension among Indians across various age-groups. Excess body weight, a critical yet modifiable risk factor for hypertension [30], varies across gender, and may explain some of the male-female differences in hypertension. But the contribution of excess body weight to the gender difference has also not been examined in studies from India, thereby limiting critical evidence in this field.

Therefore, the contribution of difference in body mass index (BMI) between males and females to these gender differences in elevated blood pressure was also examined.

As the current work deals primarily with a prevailing societal perception, we use the term “gender” instead of “sex” throughout the article, because gender refers to the socially constructed differences in the characteristics between men and women, whereas sex relates to their biological differences only [31].

## Methods

### Approach

We employed cross-sectional study design. To achieve the aim of our research, we needed a nationally representative sample of Indians representing the entire adult age spectrum. Individuals within the age range of 15–49 years came from National Family Health Survey round 4 (NFHS-4) [32], and for older age group (> 49 years) we considered the Study on Global AGEing and Adult Health (SAGE) wave 2 sample [33]. Both the surveys were conducted in 2015. The two datasets were combined (hereafter referred to as NFHS-SAGE dataset), which made up a representative sample of Indians aged 15years+. However, for our analyses we considered participants of 35 years and older from NFHS-SAGE, because hypertension is a rare event before that age [34, 35].

We repeated the analysis in the Longitudinal Ageing Study of India [36] wave 1 (hereinafter referred to as LASI) dataset for validation of our results. LASI was conducted in 2017, comprising a nationally representative sample of 45 + years old Indians and their spouses (irrespective of their age).

### Data

National Family Health Survey (NFHS) is the Indian equivalent of Demographic and Health Survey (DHS) [37], which is periodically conducted in many countries worldwide. The fourth round of NFHS collected information from a nationally representative sample of 601,509 Indian households which were selected using a multistage stratified random cluster sampling design; 699,866 women aged 15–49 years and 112,122 men aged 15–54 years were interviewed. The response rate (for both questionnaire and biomarker measurements) was 96.7% among females and 91.9% among males.

SAGE is part of a Longitudinal Survey Programme by WHO’s Multi-Country Studies unit [33]. It compiled longitudinal information on health, well-being and ageing process of the adult population of India and five other countries (China, Ghana, Mexico, Russian Federation and South Africa). The SAGE wave 2 India survey was conducted in six states, one state from each region. A multistage stratified random cluster sampling design was used to select 7118 persons aged 50 years or more (3,781 female and 3,337 male) for interview and biomarker measurement. The response rate of SAGE wave 2 was 77%.

The Longitudinal Ageing Study in India (LASI) is the Indian version of the Health and Retirement Survey(HRS) [38] geared to offer empirical evidence on indicators related to ageing and health, economic transitions, and social behaviours in later life. The wave 1 of LASI was carried out in all 29 Indian states (except Sikkim) and 6 union territories using sampling strategy and sampling frame of NFHS-4. The wave 1 of LASI interviewed a nationally representative sample of 72,250 older adults aged 45 years and above and their spouses who reside in the same household, irrespective of their age. The response rate of LASI was 87.3%. In our analysis, we have considered LASI adults aged 45 and above only, thereby excluding the spouses aged less than 45 years.

**Fig. 1:**
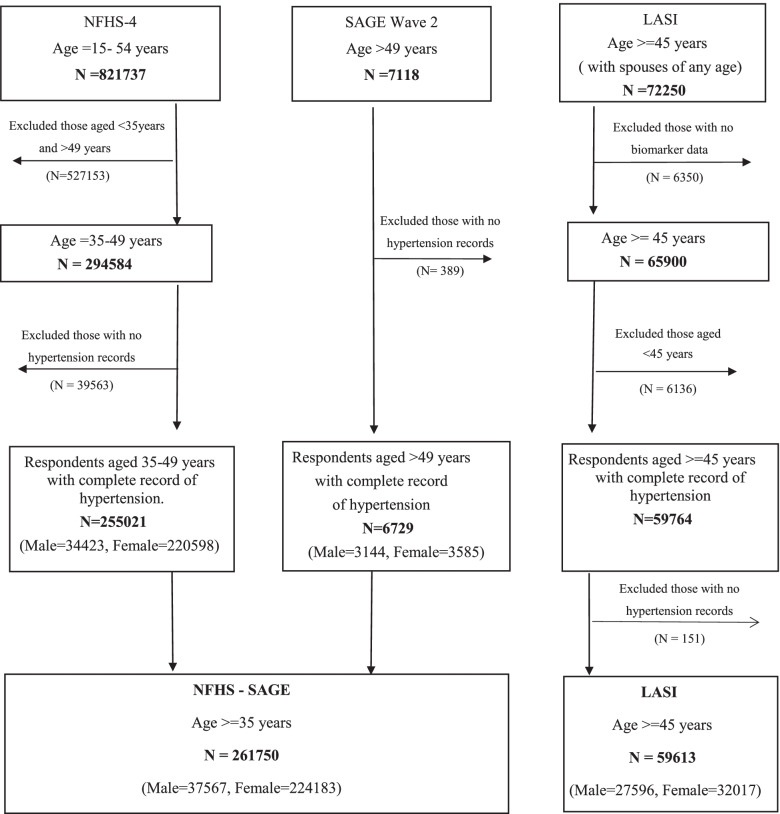
Schematic figure explaining sample–4 and SAGE wave 2 sample extraction

The NFHS-SAGE and LASI datasets that were finally included in our analyses are described in Fig. 1.

### Variables

Gender was a binary variable (Male/Female) in all the three surveys. Age was treated as a quantitative variable, later categorized into the following groups: 35–39,40–44,45–49,50–54,55–59,60–64,65–69,>70 in NFHS-SAGE dataset and 45–49,50–54,55–59,60–64,65–69,>70 in LASI datasets using a commonly used class interval of 5 years [26, 39]. However, the oldest group (≥ 70 years) comprised all respondents who were 70 years and above to ensure enough numbers in this extreme category.

Body Mass Index (BMI) was calculated as measured weight (kg) divided by measured height (m) squared. It was then dichotomized into overweight/obese (BMI > 25 kg/m^2^) and underweight /normal weight ( < = 25 kg/m^2^). Blood pressure (BP) was measured when respondents were seated and relaxed. Three BP readings were recorded with 1 min gap in between with the help of OMRON Blood Pressure Monitor (OMRON Healthcare, Hoofddorp, Netherlands) in SAGE wave 2 and NFHS-4 and two readings were recorded using Bosco wrist monitor in LASI [40]. In addition to recording blood pressure, all respondents were asked, ‘Were you told on two or more different occasions by a doctor or other health professional that you had hypertension or high blood pressure?’ If they responded “yes”, they were asked ‘To lower your blood pressure, are you taking a prescribed medicine?’ However, in NFHS-4 dataset the medicine question was asked to all respondents and not only to those answering “yes” to the diagnosis question. Consequently, NFHS-4 dataset contained few individuals who reported medication intake without a prior diagnosis of hypertension.

### Statistical analysis

The main outcome, hypertension(Y/N), was defined when average of three systolic blood pressures in case of NFHS-SAGE and average of two systolic blood pressures in case of LASI exceeded 139 and /or average of three diastolic blood pressures in case of NFHS-SAGE and average of two diastolic blood pressures in case of LASI exceeded 89 mm and/or the individual received anti-hypertensive medications [26, 41–43].

Predicted prevalence of hypertension (with 95% confidence intervals) for each age-group was estimated separately for males and females. These prevalence estimates were plotted to visualize the gender difference in hypertension in different age-groups and check whether the gender pattern reversed at any stage or not.

Age gradient of hypertension was measured using prevalence Odds Ratio (OR) from logistic regression models which included hypertension as the binary outcome and continuous measure of age as the exposure – the ORs representing by what factor the odds of hypertension increased when age increased by one year.

The modelling was carried out first in gender-pooled sample and then an age*gender interaction term was introduced in the model to examine whether the age gradients varied across gender. The OR of the interaction term represented the male female difference of age gradients in a ratio scale. The model was further adjusted for BMI (included as a continuous variable) to estimate its influence on the male female difference. We also then estimated the age gradients of hypertension separately for males and females as we found the age*gender interaction term to be statistically significant (*p* < 0.05).

We did not adjust for any co-morbidities such as diabetes and other diseases which may be associated with hypertension, because they were not relevant to our research objective.

All estimates were computed using survey weights and accounting for the complex survey design. The statistical analysis was performed using R software (version 4.2.1) and *margins* and *ggplot2* packages of R. We ensure that items of Strengthening the Reporting of Observational studies in Epidemiology (STROBE) guideline were reported wherever applicable [44].

### Sensitivity analysis

We conducted two sensitivity analyses to examine whether changing few analytical parameters led to significant divergence from the main results. First, SAGE data were collected from only 6 Indian states whereas NFHS-4 and LASI data were collected from all the 29 states and 6 Union Territories (except Sikkim) of India. As this may be a source of bias, we conducted a sensitivity analysis on a subset of LASI data containing the same six states as in SAGE, and estimated the age-wise prevalence of hypertension in males and females.

Second, in the main analysis we defined hypertension as systolic and/or diastolic blood pressure more than 139 and 89 mm of Hg respectively and/or when someone reported taking anti-hypertensive treatment [26, 41, 43]. Others have further extended the definition to include respondents who self-reported previous diagnosis of hypertension [39]. Therefore, we conducted a sensitivity analysis estimating the age-wise prevalence of hypertension in males and females using the extended definition.

## Results

The age range of respondents was 35 to 106 in NFHS-SAGE and 45 to 110 in LASI. Females made up 84% of NFHS-SAGE and 54% of LASI.

Overall, females were more likely to be overweight/obese as compared to males in both the datasets (NFHS-SAGE: 29.2% in females vs. 24.3% in males, *p* < 0.001) (LASI: 31.9% in females vs. 21.3% in males, *p* < 0.001) and this female preponderance in excess body weight were consistently observed in every age group (Additional file 1). In the younger sample, NFHS-SAGE, overall females were less likely to be hypertensive (24.1% in females vs. 29.3% in males, *p* < 0.001), whereas in older sample, LASI, overall females were slightly more predisposed to hypertension than males (46.4% in females vs. 44.7% in males, *p* < 0.001) (Table 1).

**Table 1:** NFHS-SAGE and LASI sample characteristics

	NFHS-SAGE			LASI		
	**Male**	**Female**	**Total**	**Male**	**Female**	**Total**
	(*n* = 37,567)	(*n* = 224,183)	(*n* = 261,570)	(*n* = 27,596)	(*n* = 32,017)	(*n* = 59,613)
**Age (years)**						
35–39	12,930 (13.5)	82,833 (86.5)	95,763	Not Apllicable	Not Applicable	Not Applicable
40–44	11,100 (13.5)	70,564 (86.4)	81,664	Not Applicable	Not applicable	Not Applicable
45–49	10,393 (13.3)	67,201 (86.6)	77,594	5,326 (44.3)	6,694 (55.6)	12,020
50–54	511 (37.0)	870 (63.0)	1,381	4,527 (45.4)	5,436 (54.5)	9,963
55–59	577 (43.0)	764 (56.9)	1,341	4,039 (44.1)	5,107 (55.8)	9,146
60–64	749 (49.7)	757 (50.2)	1,506	4,238 (45.5)	5,057 (54.4)	9,295
65–69	481 (50.1)	478 (49.8)	959	3,963 (49.0)	4,121 (50.9)	8,084
≥ 70	826 (53.5)	716 (46.4)	1,542	5,503 (49.5)	5,602 (50.4)	11,105
**Hypertension**						
Yes	10,991 (29.3)	53,994 (24.1)	64,985	12,323 (44.7)	14,854 (46.4)	27,177
**Body Mass Index**						
> 25 Kg/ m2	9673 (24.3)	70,771 (29.2)	80,444	5778 (21.3)	10,202 (31.9)	17,237

**Fig. 2:**
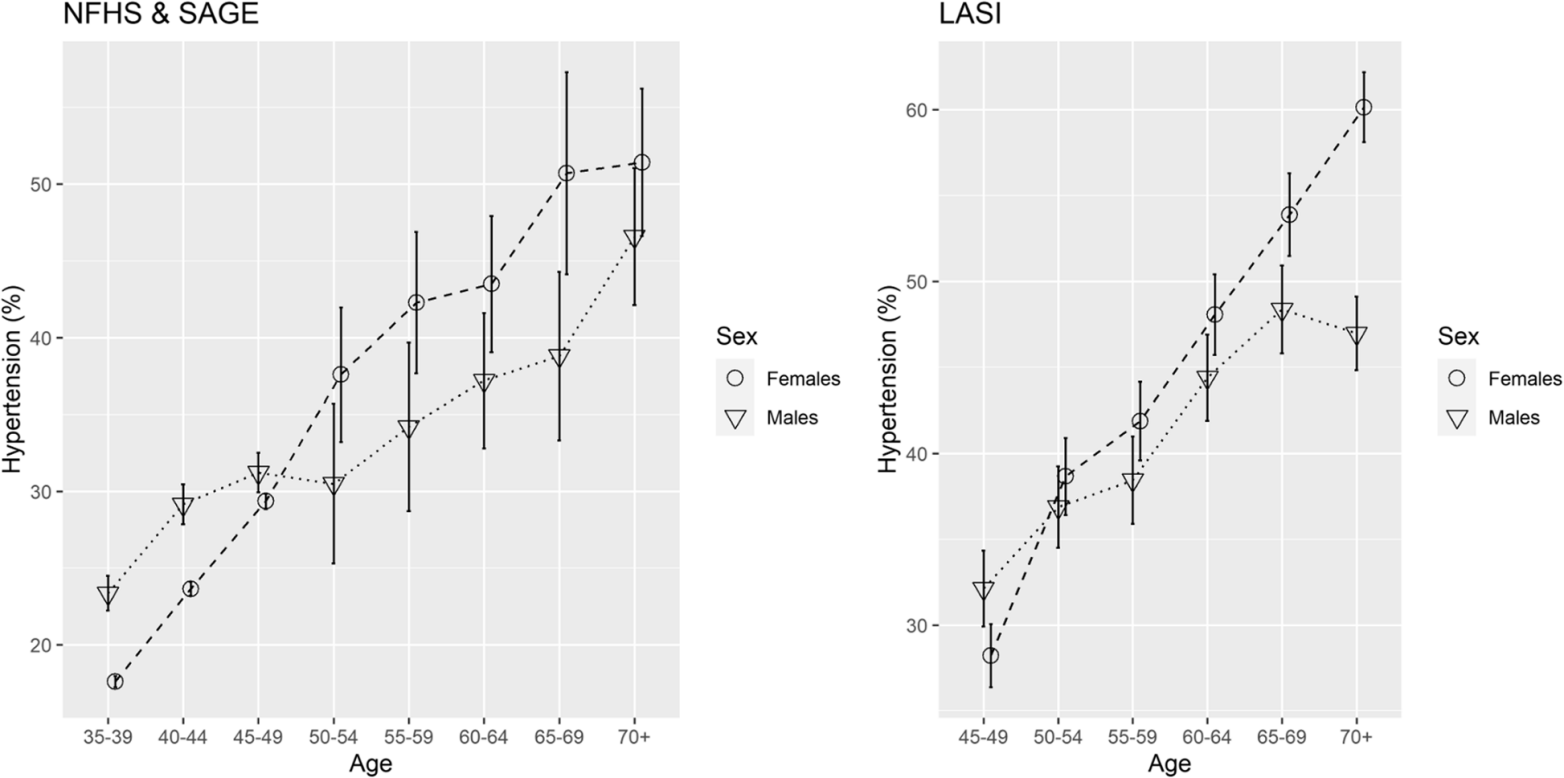
Represents the predicted probability of hypertension prevalence by sex and age in NFHS & SAGE and LASI

The prevalence of hypertension was more among males up to the age of 50 years; beyond that, in every age group, females experienced higher rates. The female-male gap in later years widened even further. For instance, prevalence in males and females were 31.23% and 29.37% in NFHS-SAGE and 32.12% and 28.21% in LASI respectively in 45–49 years age group. This changed to 30.51% and 37.61% (NFHS -SAGE) and 36.87% and 38.65% (LASI) in the 50–54 years age group – the age band when hypertension in females surpassed males (Table 2; Fig. 2).

**Table 2:** Predicted prevalence (presented as proportion of hypertensives and 95% confidence interval) of hypertension across different age groups, stratified by gender in NFHS-SAGE and LASI datasets

	NFHS-SAGE		LASI	
**Age group**	**Male**	**Female**	**Male**	**Female**
35–39	23.37 (22.25–24.50)	17.61 (17.24–17.99)	Not Applicable	Not Applicable
40–44	29.17 (27.86–30.48)	23.66 (23.20-24.12)	Not Applicable	Not Applicable
45–49	31.23 (29.95–32.52)	29.37 (28.86–29.87)	32.12 (30.14–34.11)	28.21 (25.74–30.68)
50–54	30.51 (25.31–35.72)	37.61 (33.24–41.98)	36.87 (32.41–41.34)	38.65 (34.95–42.35)
55–59	34.21 (28.74–39.69)	42.29 (37.68–46.90)	38.44 (34.44–42.43)	41.88 (39.36–44.39)
60–64	37.21 (32.81–41.62)	43.50 (39.05–47.94)	44.40 (41.81–46.98)	48.06 (45.94–50.17)
65–69	38.81 (33.34–44.28)	50.71 (44.13–57.29)	48.36 (45.90-50.82)	53.88 (50.05–57.71)
> 70	46.59 (42.12–51.06)	51.43 (46.63–56.23)	46.96 (44.54–49.38)	60.14 (57.39–62.88)

In NFHS-SAGE, the age gradient of hypertension, expressed by OR, was 1.04 (95% confidence interval: 1.04,1.04) in the gender-pooled dataset, 1.02 (1.02,1.02) in males and was 1.05 (1.05, 1.06) in females. The male: female ratio of the gradients was 0.96 (0.96, 0.97), which changed marginally to 0.97 (0.97, 0.97) after adjustment with BMI, signifying that the male female difference was hardly explained by differential distribution of BMI across gender. In LASI, the age gradient was 1.03 (1.02, 1.03) in the pooled dataset, 1.01 (1.01,1.02) in males and 1.04 (1.03,1.04) in females, the male: female ratio of the gradients being 0.97 (0.97,0.98). Again, the male: female ratio hardly changed after adjustment with BMI (Table 3).

Two sensitivity analyse were carried out, first one on a subset of LASI data with only six Indian states as in the SAGE dataset, and second one using an extended definition of hypertension. In both the occasions, reversal of the gender pattern occurred between 50 and 54 years, similar to our main result (Additional files 2 and 3),

**Table 3:** Gender differentials in age gradient of hypertension in NFHS-SAGE and LASI datasets

		NFHS-SAGE Odds Ratio (95% CI), *p* value	LASI Odds Ratio (95% CI), *p* value
**Age gradient of hypertension**			
Pooled		1.04(1.04–1.04),<0.001	1.03(1.02–1.03), < 0.001
Male		1.02(1.02–1.02),<0.001	1.01( 1.01–1.02),<0.001
Female		1.05(1.05–1.06),<0.001	1.04( 1.03–1.04),<0.001
**Ratio of male:female age gradients of hypertension**			
Unadjusted		0.96(0.96–0.97),<0.001	0.97( 0.97–0.98),<0.001
Adjusted with BMI		0.97(0.97–0.97),<0.001	0.97( 0.96–0.97),<0.001

## Discussion

We found hypertension to be more prevalent in Indian males in the age group of 35–50 years, but after 50 years of age hypertension became more prevalent in Indian females. In both the datasets that we analyzed, the reversal of the gender pattern occurred between 50 and 54 years. Despite females being more likely to be overweight/ obese than males at all stages of their lives, the gender difference in excess weight hardly explained the gender pattern of hypertension and its changes over age.

Most of the previous studies from India only looked at the overall (age-pooled) gender difference in prevalence of hypertension [27, 45, 46] and that too mostly in younger populations [27, 29], but none looked at the differences across all adult age groups. Therefore, almost all Indian studies reported Indian males to be more prone to hypertension than females [7, 27]. Only one study comprising a large yet volunteer sample, recorded blood pressure of the participants in a mass campaign mode. This was the only previous Indian study to our knowledge to explore the gender difference in hypertension across entire adult life. Even their results showed more males having hypertension in every age group, except in those aged ≥ 65 years, [7]which had equal proportion of males and females suffering from the condition. Therefore, to our knowledge, ours is the first study from India to report conclusively that after 50 years of age, the prevalence of hypertension among females surpasses that of males and the female-male gap continues to increase beyond that age.

We found gender difference in hypertension and its changing pattern with age in India to be similar to the patterns seen in high-income western nations, despite their significant socio-cultural differences [19, 24, 28, 47]. This underscores that it may not be the socio-cultural factors but biology of ageing, such as depletion of female sex hormones after menopause [22, 48–50], which may be the primary driver of excess hypertension among females in later life. Other studies and reports from the same datasets we analyzed [39, 40] have shown that Indian females had greater rate of diagnosis of their hypertension, more access to treatment and better control than their age-matched male counterparts. Therefore, it will not be incorrect to conclude that the higher prevalence of hypertension that we observed in Indian females in later life is unlikely to be due to differential access to diagnosis and treatment services.

The most important contribution of our study is to the deconstruction of the popular misconception that females, especially in India, are less predisposed to cardiovascular diseases and hypertension. This myth exists worldwide [13], but it may exist in an even exaggerated form in India, because of the prevailing idea of Indian socio-cultural “exceptionalism”, which considers the lifestyle of Indian women to be traditional, hence, different from their western counterparts; therefore, Indian women may be even likely to be affected by chronic lifestyle-related diseases like hypertension [12, 46, 51–53]. Even popular media such as movies and TV shows, as well as market (advertisement for healthcare services and products) often portray only males [12] as victims of cardiovascular outcomes. In this backdrop, our study provides strong evidence to demystify the myth. Therefore, our results also have significant policy implications in terms of deciding the contents of CVD-related health messages and guiding relevant health promotion initiatives in this field, which should provide adequate if not greater priority to Indian women. Although evidence shows women hypertensives are diagnosed and treated in greater numbers than men, but approximately more than half of the hypertensives in India remain undiagnosed regardless of their gender [39, 54]. So the women who are diagnosed are more likely to be from the privileged section as underdiagnosis is rampant among poor, rural and less educated [55].Currently, Ayushman Bharat is being rolled-out as the flagship health assurance programme of government of India for providing free healthcare services to the poorest 40% Indians, The programme, through newly developed Health and Wellness Centers is gearing up to provide free primary care to large section of Indians, with a special focus on non-communicable diseases and their risk factors [39, 54, 56]. It plans to cover a large section which otherwise remains currently underserved. The prevailing popular misconception may misdirect the rapid expansion of its service coverage and females are at a risk of being left behind. Therefore, gender sensitivity has to be strongly incorporated in the programme, so that Indian females in their later life are appropriately diagnosed and treated.

Our study has few limitations also. Cross-sectional data was used which may bias the age gradient of hypertension by introducing “birth cohort” effect [57]. However, longitudinal studies from the western nations also reported similar gender patterns [50, 58], which validates our findings. The second likely limitation is that the combined dataset of NFHS-SAGE has disproportionately large numbers of younger participants and females. But that hardly compromises our conclusions, except leading to widening of confidence intervals of the prevalence estimates in the older age group in the NFHS-SAGE sample due to smaller sample size of SAGE wave 2 as compared to NFHS-4. One can also notice consistently higher hypertension rates in the LASI sample than contemporary SAGE wave 2 sample (these two studies were conducted only two years apart). This is because in SAGE wave 2, prevalence was estimated from only six Indian states that did not include many prosperous hypertension-burdened Indian states such as Kerala, Punjab, Haryana, Delhi and others, whereas LASI covered all the 29 Indian states (except Sikkim).Consequently, hypertension estimates from LASI were on the higher side. However, as we only aimed to estimate the male-female difference in hypertension in different stages of life and not national prevalence of hypertension, the differences between SAGE and LASI samples did not affect our conclusions, which the results of the sensitivity analysis conducted on a truncated LASI sample also corroborated.

The strength of our study lies in the use of three large national datasets and replication of the results of the first analysis in a second dataset, thereby substantially strengthening the conclusions.

## Conclusions

To conclude our study showed that Indian females are more likely to be hypertensive than age-matched males after the 50th year of their lives. Therefore, CVD and hypertension-related health promotion messages and primary health care policies aimed at these pathologies should prioritize women in later life. Also, there is a knowledge gap regarding the healthcare access factors that may underlie higher female preponderance in later life, which needs comprehensive exploration in future.

## Supplementary Information

**Additional file 1.**

**Additional file 2.**

**Additional file 3.**

## Data Availability

The datasets generated and/or analyzed during this work are accessible in the International Institute for Population Sciences’ repository in Mumbai, India, and can be viewed freely available in public domain through http://iipsindia.org.

## Abbreviations

BMI: body mass index
NFHS: National Family Health Survey
LASI: Longitudinal Ageing Study of India
SAGE: Study of Ageing and Health
ORs: Odds Ratios
NCD: Non-Communicable Diseases
LMICs: Low- and Middle-Income Countries
CVD: Cardiovascular Diseases
Y/N: Yes/No
Kg: kilogram
m: Meter
Cm: centimetres

## References

[1] Noncommunicable diseases: Mortality [Internet]. [cited 2022 Apr 7]. Available from: https://www.who.int/data/gho/data/themes/topics/topic-details/GHO/ncd-mortality

[2] Bromfield S, Muntner P (2013). High Blood Pressure: The Leading Global Burden of Disease Risk Factor and the Need for Worldwide Prevention Programs. Curr Hypertens Rep.

[3] Mills KT, Bundy JD, Kelly TN, Reed JE, Kearney PM, Reynolds K (2016). Global Disparities of Hypertension Prevalence and Control: A Systematic Analysis of Population-Based Studies From 90 Countries. Circulation.

[4] Hogerzeil HV, Liberman J, Wirtz VJ, Kishore SP, Selvaraj S, Kiddell-Monroe R (2013). Promotion of access to essential medicines for non-communicable diseases: practical implications of the UN political declaration. Lancet.

[5] Islam SMS, Purnat TD, Phuong NTA, Mwingira U, Schacht K, Fröschl G. Non-Communicable Diseases (NCDs) in developing countries: a symposium report. Globalization Health. 2014;10(1):81. 10.1186/s12992-014-0081-9 PMC4267750 25498459

[6] Miranda JJ, Kinra S, Casas JP, Smith GD, Ebrahim S. Non-communicable diseases in low- and middle-income countries: context, determinants and health policy. Trop Med Int Health. 2008;13(10):1225–34. 10.1111/j.1365-3156.2008.02116.x PMC2687091 18937743

[7] Ramakrishnan S, Zachariah G, Gupta K, Shivkumar Rao J, Mohanan PP, Venugopal K (2019). Prevalence of hypertension among Indian adults: Results from the great India blood pressure survey. Indian Heart J..

[8] Kumar K, Misra S (2021). Sex differences in prevalence and risk factors of hypertension in India: Evidence from the National Family Health Survey-4. PLoS One.

[9] Hypertension in India - PubMed [Internet]. [cited 2022 Mar 17]. Available from: https://pubmed.ncbi.nlm.nih.gov/24781507/

[10] Jahan Y, Moriyama M, Rahman MM, Kazawa K, Mizukawa M, Rahman A (2020). Disease perception and experiences among rural Bangladeshi hypertensive women: A qualitative approach. Health Promot Perspect.

[11] Kusuma YS. Perceptions on hypertension among migrants in Delhi, India: a qualitative study. BMC Public Health. 2009;9(1):267. 10.1186/1471-2458-9-267 PMC2725039 19638224

[12] Kaptein AA, van der Meer PB, Florijn BW, Hilt AD, Murray M, Schalij MJ. Heart in art: cardiovascular diseases in novels, films, and paintings. Philosophy Ethics Hum Med. 2020;15(1):2. 10.1186/s13010-020-0086-3 PMC7017445 32050992

[13] Woodward M (2019). Cardiovascular Disease and the Female Disadvantage. Int J Environ Res Public Health.

[14] McDonnell LA, Pipe AL, Westcott C, Perron S, Younger-Lewis D, Elias N (2014). Perceived vs actual knowledge and risk of heart disease in women: findings from a Canadian survey on heart health awareness, attitudes, and lifestyle. Can J Cardiol.

[15] Hypertension symptoms in women often mistaken for menopause. [cited 2022 Apr 7]. Available from: https://www.escardio.org/The-ESC/Press-Office/Press-releases/Hypertension-symptoms-in-women-often-mistaken-for-menopause, https://www.escardio.org/The-ESC/Press-Office/Press-releases/Hypertension-symptoms-in-women-often-mistaken-for-menopause

[16] Correction: Heart disease and stroke statistics-2017 update: A report from the American Heart Association (Circulation (2017) 135 (e146-e603) DOI: 10.1161/CIR.0000000000000485). Vol. 136, Circulation. Lippincott Williams and Wilkins; 2017. p. e196. 10.1161/CIR.0000000000000485 PMC5408160 28122885

[17] Roger VL, Go AS, Lloyd-Jones DM, Adams RJ, Berry JD, Brown TM, et al. Heart disease and stroke statistics-2011 update: A report from the American Heart Association. Circulation. 2011;123(4):e18. 10.1161/CIR.0b013e3182009701 PMC4418670 21160056

[18] Oliveira IM, Duarte YADO, Zanetta DMT. Prevalence of Systemic Arterial Hypertension Diagnosed, Undiagnosed, and Uncontrolled in Elderly Population: SABE Study. J Aging Res. 2019;2019. 10.1155/2019/3671869 PMC6745120 31565434

[19] Doumas M, Papademetriou V, Faselis C, Kokkinos P (2013). Gender differences in hypertension: myths and reality. Curr Hypertens Rep.

[20] Martins D, Nelson K, Pan D, Tareen N, Norris K (2001). The effect of gender on age-related blood pressure changes and the prevalence of isolated systolic hypertension among older adults: data from NHANES III. J Gend Specif Med.

[21] Hermida RC, Ayala DE, Mojón A, Fontao MJ, Chayán L, Fernández JR (2013). Differences between men and women in ambulatory blood pressure thresholds for diagnosis of hypertension based on cardiovascular outcomes. Chronobiol Int..

[22] Gierach GL, Johnson BD, Bairey Merz CN, Kelsey SF, Bittner V, Olson MB, et al. Hypertension, menopause, and coronary artery disease risk in the Women’s Ischemia Syndrome Evaluation (WISE) Study. J Am Coll Cardiol. 2006;47(3 Suppl):S50-58. 10.1016/j.jacc.2005.02.099 16458172

[23] Wang W, Lee ET, Fabsitz RR, Devereux R, Best L, Welty TK (2006). A longitudinal study of hypertension risk factors and their relation to cardiovascular disease: the Strong Heart Study. Hypertension.

[24] Pearson JD, Morrell CH, Brant LJ, Landis PK, Fleg JL (1997). Age-associated changes in blood pressure in a longitudinal study of healthy men and women. J Gerontol A Biol Sci Med Sci.

[25] Kannel WB, Gordan T (1978). Evaluation of cardiovascular risk in the elderly: the Framingham study. Bull N Y Acad Med.

[26] Lloyd-Sherlock P, Beard J, Minicuci N, Ebrahim S, Chatterji S (2014). Hypertension among older adults in low- and middle-income countries: prevalence, awareness and control. Int J Epidemiol.

[27] Ghosh S, Mukhopadhyay S, Barik A. Sex differences in the risk profile of hypertension: a cross-sectional study. BMJ Open. 2016;6(7):e010085. 10.1136/bmjopen-2015-010085 PMC4964242 27466234

[28] Gupta R (2004). Trends in hypertension epidemiology in India. J Hum Hypertens.

[29] Busingye D, Arabshahi S, Evans RG, Riddell MA, Srikanth VK, Kartik K (2019). Knowledge of risk factors for hypertension in a rural Indian population. Heart Asia.

[30] Shihab HM, Meoni LA, Audrey S;, Chu Y, Wang NY, Ford DE, et al. Body Mass Index and Risk of Incident Hypertension Over the Life Course The Johns Hopkins Precursors Study. 2012; 10.1161/CIRCULATIONAHA.112.117333 PMC3743236 23151344

[31] Nielsen MW, Stefanick ML, Peragine D, Neilands TB, Ioannidis JPA, Pilote L, et al. Gender-related variables for health research. Biology of Sex Differences. 2021;12(1):23. 10.1186/s13293-021-00366-3 PMC7898259 33618769

[32] NATIONAL FAMILY HEALTH SURVEY (NFHS-4) 2015-16 INDIA. 2017.

[33] SAGE Wave-2 | International Institute for Population Sciences (IIPS) [Internet]. [cited 2022 May 8]. Available from: https://iipsindia.ac.in/content/SAGE-wave-2

[34] Hypertension in Adults Across the Age Spectrum: Current Outcomes and Control in the Community | Hypertension | JAMA | JAMA Network [Internet]. [cited 2022 May 8]. Available from: https://jamanetwork.com/journals/jama/fullarticle/201294 10.1001/jama.294.4.466 16046653

[35] Hypertension [Internet]. [cited 2022 May 8]. Available from: https://www.who.int/news-room/fact-sheets/detail/hypertension

[36] LASI. Longitudinal Ageing Study in India (LASI) INDIA Wave-1. 2017;

[37] Fabic MS, Choi YJ, Bird S (2012). A systematic review of Demographic and Health Surveys: data availability and utilization for research. Bulletin of the World Health Organization.

[38] Fisher GG, Ryan LH (2018). Overview of the health and retirement study and introduction to the special issue. Work, Aging and Retirement.

[39] Prenissl J, Manne-Goehler J, Jaacks LM, Prabhakaran D, Awasthi A, Bischops AC (2019). Hypertension screening, awareness, treatment, and control in India: A nationally representative cross-sectional study among individuals aged 15 to 49 years. PLoS Med.

[40] LASI_India_Report_2020_compressed.pdf [Internet]. [cited 2022 Jul 6]. Available from: https://www.iipsindia.ac.in/sites/default/files/LASI_India_Report_2020_compressed.pdf

[41] Prevalence and associated risk factors of hypertension among persons aged 15–49 in India: a cross-sectional study | BMJ Open [Internet]. [cited 2022 Jul 6]. Available from: https://bmjopen.bmj.com/content/9/12/e029714.long 10.1136/bmjopen-2019-029714 PMC6937064 31848161

[42] Hu P, Lee J. Harmonization of Cross-National Studies of Aging to the Health and Retirement Study: Chronic Medical Conditions [Internet]. RAND Corporation; 2012 Feb [cited 2022 Jul 6]. Available from: https://www.rand.org/pubs/working_papers/WR861z1.html

[43] Moser KA, Agrawal S, Davey Smith G, Ebrahim S (2014). Socio-demographic inequalities in the prevalence, diagnosis and management of hypertension in India: analysis of nationally-representative survey data. PLoS One.

[44] Cuschieri S (2019). The STROBE guidelines. Saudi J Anaesth.

[45] Reddy BM, Ganguly E, Sharma PK (2018). Hypertension and its Correlates in the Oldest Old Population Aged 80 Years and Above in Urban South India. J Gerontol Geriatr Res.

[46] Gao Z, Chen Z, Sun A, Deng X (2019). Gender differences in cardiovascular disease. Medicine in Novel Technology and Devices.

[47] Zhou B, Bentham J, Cesare MD, Bixby H, Danaei G, Cowan MJ (2017). Worldwide trends in blood pressure from 1975 to 2015: a pooled analysis of 1479 population-based measurement studies with 19·1 million participants. Lancet.

[48] Wenger NK, Arnold A, Bairey Merz CN, Cooper-DeHoff RM, Ferdinand KC, Fleg JL (2018). Hypertension Across a Woman’s Life Cycle. J Am Coll Cardiol.

[49] Coylewright M, Reckelhoff JF, Ouyang P. Menopause and Hypertension. Hypertension. 2008;51(4):952–9. 10.1161/HYPERTENSIONAHA.107.105742 18259027

[50] Reckelhoff JF. Gender differences in the regulation of blood pressure. Hypertension. 2001;37(5):1199–208. 10.1161/01.hyp.37.5.1199 11358929

[51] Weidner G (2000). Why do men get more heart disease than women? An international perspective. J Am Coll Health.

[52] Grundtvig M, Hagen TP, German M, Reikvam A (2009). Sex-based differences in premature first myocardial infarction caused by smoking: twice as many years lost by women as by men. Eur J Cardiovasc Prev Rehabil..

[53] Prescott E, Hippe M, Schnohr P, Hein HO, Vestbo J (1998). Smoking and risk of myocardial infarction in women and men: longitudinal population study. BMJ.

[54] Basu S, Millett C (2013). Social epidemiology of hypertension in middle-income countries: determinants of prevalence, diagnosis, treatment, and control in the WHO SAGE study. Hypertension.

[55] Karmakar N, Nag K, Saha I, Parthasarathi R, Patra M, Sinha R (2018). Awareness, treatment, and control of hypertension among adult population in a rural community of Singur block, Hooghly District, West Bengal. J Educ Health Promot.

[56] Angell BJ, Prinja S, Gupt A, Jha V, Jan S (2019). The Ayushman Bharat Pradhan Mantri Jan Arogya Yojana and the path to universal health coverage in India: Overcoming the challenges of stewardship and governance. PLoS Med.

[57] Canova C, Cantarutti A (2020). Population-Based Birth Cohort Studies in Epidemiology. Int J Environ Res Public Health.

[58] Roger VL, Go AS, Lloyd-Jones DM, Adams RJ, Berry JD, Brown TM, et al. Heart disease and stroke statistics–2011 update: a report from the American Heart Association. Circulation. 2011;123(4):e18–209. 10.1161/CIR.0b013e3182009701 PMC4418670 21160056

