# Supplementary material for: Gender difference in prevalence of hypertension among Indians across various age-groups: a report from multiple nationally representative samples
Source: BMC Public Health. 2022 Aug 10;22:1524. doi: 10.1186/s12889-022-13949-5 (PMC9364494; doi:10.1186/s12889-022-13949-5)
Supplement: Supplementary file 1 — Additional file 1. [file 12889_2022_13949_MOESM1_ESM.docx]

**Table S1: Predicted prevalence (presented as proportion of Overweight and 95% confidence interval) of Overweight across different age groups, stratified by gender in NFHS-SAGE and LASI datasets.**

|  | **NFHS-SAGE** | | **LASI** | | |
| --- | --- | --- | --- | --- | --- |
| **Age group** | **Male** | **Female** | **Male** | **Female** | |
| 35-39 | 26.32(25.58-27.07) | 29.27(28.97-29.56) |  | | |
| 40-44 | 27.74(26.93-28.55) | 32.81(32.47-33.14) |  |  |  |
| 45-49 | 27.58(26.75-28.41) | 34.16(33.82-34.51) | 25.23(23.17-27.29) | | 37.39(35.40-39.37) |
| 50-54 | 20.06(17.12-23.01) | 24.75(22.25-27.25) | 28.05(25.83-30.27) | | 38.04(35.80-40.28) |
| 55-59 | 20.63(17.83-23.43) | 27.34(24.57-30.10) | 22.68(20.47-24.88) | | 36.17(33.95-38.39) |
| 60-64 | 13.78(11.64-15.92) | 21.17(18.59-23.76) | 21.44(19.36-23.51) | | 31.16(28.97-33.34) |
| 65-69 | 11.48(09.03-13.93) | 19.98(16.92-23.03) | 18.59(16.59-20.58) | | 29.54(27.33-31.75) |
| >70 | 11.99(10.03-13.95) | 15.36(12.99-17.73) | 14.05(12.54-15.56) | | 19.89(18.21-21.58) |
