# Supplementary material for: Gender difference in prevalence of hypertension among Indians across various age-groups: a report from multiple nationally representative samples
Source: BMC Public Health. 2022 Aug 10;22:1524. doi: 10.1186/s12889-022-13949-5 (PMC9364494; doi:10.1186/s12889-022-13949-5)
Supplement: Supplementary file 2 — Additional file 2. [file 12889_2022_13949_MOESM2_ESM.docx]

**Table S2.** **Sensitivity analysis of predicted prevalence (presented as proportion of hypertensives and 95% confidence interval) of hypertension across different age groups, stratified by gender in six states of LASI datasets**

|  | **LASI** | |
| --- | --- | --- |
| **Age group** | **Male** | **Female** |
| 45-49 | 30.19(33.45-26.93) | 27.14(29.81-24.48) |
| 50-54 | 36.11(39.53-32.70) | 39.40(42.72-36.07) |
| 55-59 | 36.77(40.35-33.20) | 39.57(42.85-36.28) |
| 60-64 | 42.69(46.40-38.98) | 48.90(52.53-45.27) |
| 65-69 | 48.50(52.37-44.63) | 55.12(58.52-51.71) |
| >70 | 46.85(50.01-43.70) | 61.87(64.81-58.93) |
