# Supplementary material for: Gender difference in prevalence of hypertension among Indians across various age-groups: a report from multiple nationally representative samples
Source: BMC Public Health. 2022 Aug 10;22:1524. doi: 10.1186/s12889-022-13949-5 (PMC9364494; doi:10.1186/s12889-022-13949-5)
Supplement: Supplementary file 3 — Additional file 3. [file 12889_2022_13949_MOESM3_ESM.docx]

**Table S3. Sensitivity analysis of predicted prevalence of hypertension (using extended definition of hypertension) across different age groups stratified by gender in NFHS-SAGE and LASI datasets.**

|  | **NFHS-SAGE** | | **LASI** | | |
| --- | --- | --- | --- | --- | --- |
| **Age group** | **Male** | **Female** | **Male** | **Female** | |
| 35-39 | 28.49(27.71-29.28) | 24.28(23.99-24.57) |  | | |
| 40-44 | 33.27(32.39-34.15) | 29.98(29.65-30.32) |  |  |  |
| 45-49 | 36.25(35.33-37.17) | 35.79(35.43-36.15) | 35.64(33.37-37.91) | | 34.50(32.55-36.45) |
| 50-54 | 32.01(29.41-36.21) | 40.53(37.71-43.34) | 42.32(39.89-44.76) | | 45.07(42.78-47.35) |
| 55-59 | 37.25(33.94-40.56) | 45.03(41.96-48.09) | 42.16(39.58-44.75) | | 47.47(45.16-49.77) |
| 60-64 | 40.51(37.51-43.51) | 47.94(44.82-51.05) | 47.64(45.12-50.16) | | 53.49(51.14-55.83) |
| 65-69 | 42.57(38.82-46.31) | 55.19(51.46-58.92) | 51.71(49.16-54.26) | | 58.88(56.51-61.26) |
| >70 | 50.70(47.78-53.62) | 54.89(51.79-57.99) | 50.64(48.50-52.79) | | 63.86(61.86-65.85) |
